# Impact of maternal body mass index and gestational comorbidities on the birth prevalence of orofacial clefts in the Japan Environment and Children’s Study

**DOI:** 10.1265/ehpm.25-00205

**Published:** 2025-11-01

**Authors:** Shinobu Tsuchiya, Masahiro Tsuchiya, Haruki Momma, Masatoshi Saito, Chiharu Ota, Kaoru Igarashi

**Affiliations:** 1 Department of Orthodontics and Speech Therapy for Craniofacial Anomalies, Tohoku University Hospital, Sendai, Miyagi, Japan; 2 Division of Craniofacial Anomalies, Tohoku University Graduate School of Dentistry, Sendai, Miyagi, Japan; 3 Department of Nursing, Tohoku Fukushi University, Sendai, Miyagi, Japan; 4 Laboratory of Well-Being, Center for Physical Activity Research, National Institute of Health and Nutrition, National Institutes of Biomedical Innovation, Health and Nutrition, Settsu, Osaka, Japan; 5 Department of Obstetrics and Gynecology, Tohoku University Graduate School of Medicine, Sendai, Miyagi, Japan; 6 Department of Development and Environmental Medicine, Tohoku University Graduate School of Medicine, Sendai, Miyagi, Japan; 7 Department of Pediatrics, Tohoku University Hospital, Sendai, Miyagi, Japan

**Keywords:** Cleft lip and palate, Body mass index, Gestational hypertension, Gestational diabetes, Birth cohort

## Abstract

**Background:**

An increased prevalence of cleft lip and/or palate (CL/P), a major congenital anomaly, has been observed in the offspring of women with elevated body mass index (BMI) before pregnancy. Likewise, gestational comorbidities, such as hypertension and diabetes mellitus, also increase the risk of CL/P; however, the risk linked to the coexistence of these conditions in women with higher BMI on birth prevalence of CL/P remains unclear. This study focused on the combined effects of a high BMI before pregnancy and gestational comorbidities on the birth prevalence of CL/P.

**Methods:**

Among 98,373 live births from the Japan Environment and Children’s Study (JECS), a nationwide birth cohort, 255 mothers of infants with CL/P (74, 112, and 69 infants born with cleft lip, cleft lip and palate, and isolated cleft palate, respectively) were included in the analyses. The association of CL/P birth prevalence with pre-pregnancy BMI and gestational comorbidities (hypertension and diabetes) was examined using multivariate logistic regression analyses after multiple imputations, with adjustments for several maternal (age at delivery, smoking habits, and alcohol intake) and child-related (sex and prevalence of other congenital diseases) variables, obtained through medical record transcriptions and self-reports on JECS transcription forms.

**Results:**

Higher prevalence rates of overweight, gestational hypertension, and gestational diabetes mellitus were found in mothers of infants with CL/P (16.1%, 6.3%, and 4.7%, respectively) than in the control group (10.4%, 3.1%, and 3.1%, respectively). The odds ratio [95% confidence interval] for childbirth with CL/P was increased in mothers with high BMI before pregnancy (1.58 [1.11–2.24]). Furthermore, gestational hypertension and diabetes coexisting with high BMI additionally increased the odds ratios for childbirth with CL/P (2.91 [1.28–6.61] and 2.12 [0.87–5.19], respectively).

**Conclusion:**

High maternal BMI, particularly when accompanied by gestational hypertension, was significantly associated with an increased prevalence of childbirth with CL/P.

**Supplementary information:**

The online version contains supplementary material available at https://doi.org/10.1265/ehpm.25-00205.

## Background

Cleft lip and/or cleft palate (CL/P), which generally includes cleft lip with palate (CLP), isolated cleft lip (CL), and isolated cleft palate (CP) [1], are among the most common birth defects, occurring in approximately 1 in 500–700 births [2–4]. The prevalence of CL/P varies considerably across geographic regions and racial backgrounds. In Japan, the rate is notably higher than the global average, ranging from 14.4 to 24.8 cases per 10,000 births [2, 5, 6]. CL/P is a complex, multifactorial disease caused by a combination of social, environmental, and genetic factors that result in fusion failure during craniofacial embryogenesis [4]. As parental age over 40 years [7] and maternal excessive smoking and drinking, which are recognized teratogens that increase the risk of CL/P prevalence in meta-analyses [8–10], lower socioeconomic statuses have been reported as a confounder increasing the CL/P prevalence in infants [11]. Regarding maternal metabolic abnormalities, maternal obesity has been linked to an increased prevalence of CL/P [12–17] and other congenital anomalies, such as congenital heart defects and spina bifida [14, 17].

Overweight (body mass index [BMI] ≥ 25.0 kg/m^2^) and obesity (BMI ≥ 30 kg/m^2^) are prevalent among women of reproductive age worldwide, with 44% of women aged ≥18 years classified as overweight and 16% as obese [18]. Maternal obesity increases the risks for mothers, including the prevalence of gestational hypertension, diabetes mellitus, and delivery complications. It also heightens risks for offspring, such as preterm birth, macrosomia, and perinatal complications, including CL/P [16, 17, 19–22]. In particular, gestational hypertension and diabetes mellitus, although still controversial, have been reported to independently increase the risk of CL/P in offspring [16, 23–25]. Thus, the causal relationship between maternal obesity and the increased prevalence of CL/P is further complicated by various physiological, genetic, and environmental factors. Notably, Kutbi et al. [16] summarized case-control studies from a large international dataset and showed that both obese and underweight mothers were associated with an increased risk of CL/P in their offspring, although findings regarding the association between underweight and CL/P prevalence have been inconsistent [13–16]. Among Japanese women, 16% of women are underweight at the start of their pregnancy [26, 27]. Because Japanese women who are overweight, especially those classified as obese, are fewer, while underweight women are more common than in the global population [26], metabolic abnormalities, including being underweight, may contribute to an increased prevalence of CL/P in the Japanese population. However, the impact of maternal metabolic abnormalities on the risk of CL/P in offspring remains unclear.

The Japan Environment and Children’s Study (JECS), a nationwide prospective survey of children’s health in Japan, has collected data on the health status and sociodemographic characteristics of approximately 100,000 mother–child dyads. This dataset includes information on maternal BMI, gestational disorders such as hypertension and diabetes mellitus [28, 29], and the birth prevalence of CL/P [5, 30]. Using these data, this study aimed to conduct a secondary analysis to investigate the impact of metabolic abnormalities, specifically pre-pregnancy BMI, and gestational hypertension and diabetes mellitus, on the birth prevalence of CL/P in Japanese offspring.

## Methods

### Data collection and participants

**Fig. 1:**
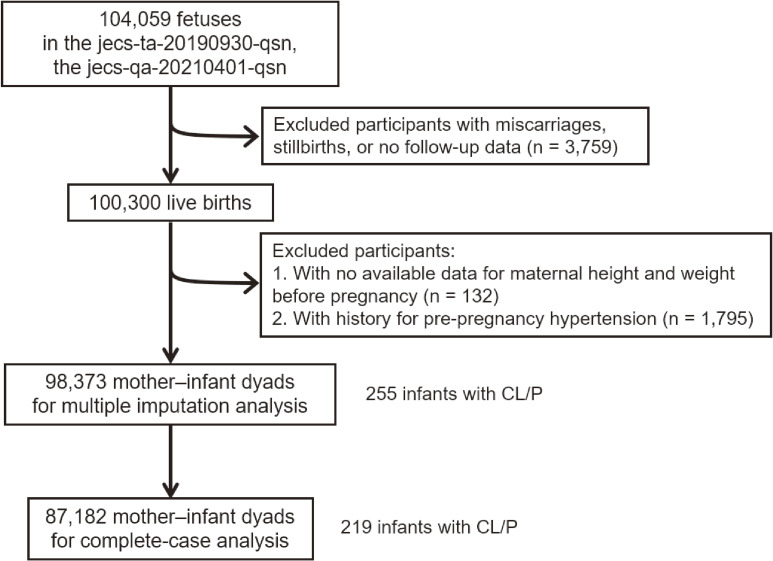
Flowchart of participant selection from Japan Environment and Children’s Study dataset.

This prospective cohort study was conducted in accordance with the Declaration of Helsinki (1975, revised in 2008). We used anonymized data from the jecs-ta-20190930-qsn and jecs-qa-20210401-qsn datasets, released in October 2019 and April 2021, respectively. The JECS protocol, published elsewhere [31, 32], was reviewed and approved by the Ministry of the Environment’s Institutional Review Board on Epidemiological Studies and the Ethics Committees of all participating institutions (no. 100910001). The aim and procedures of the study were explained to all pregnant women during early pregnancy visits in Co-operating Healthcare Providers or local government offices between January 2011 and March 2014. After obtaining written informed consent, participants completed self-administered questionnaires. Then, they underwent medical assessments by physicians, midwives, nurses, and/or Research Co-ordinators at delivery and 1 month postpartum. Details regarding data processing, validation, and verification of the perinatal assessment have been described previously [31, 32]. We enrolled 104,059 fetuses from 15 Regional Centres in the JECS. Among these pregnancies, 3,759 (3.6%) resulted in miscarriage, stillbirth, or they were lost to follow-up, and data on pre-pregnancy BMI were unavailable for 132 participants. Furthermore, 1,795 participants were excluded because of pre-pregnancy hypertension. After these exclusions, the final sample comprised 98,373 live births (Fig. 1).

### Pre-pregnancy maternal BMI (primary exposure measures)

Maternal BMI before pregnancy was calculated using body weight and height (kg/m^2^) according to the World Health Organization standards and criteria [18]. Participants were categorized into three groups: underweight (BMI < 18.5 kg/m^2^), normal weight (18.5 to <25.0 kg/m^2^), and overweight (≥25.0 kg/m^2^). It should be noted that obese participants (pre-pregnancy BMI > 30.0 kg/m^2^) were included in the overweight group owing to the small sample size and the typical BMI distribution in the general population of Japanese women [26, 27].

### Gestational hypertension and diabetes mellitus (secondary exposure measures)

The prevalence rates of gestational hypertension and diabetes mellitus were determined using medical records transcription at delivery and 1 month postpartum, as recorded on JECS transcription forms [28, 29]. Physicians, midwives, nurses, and/or Research Co-ordinators marked the corresponding checkboxes when gestational comorbidities were diagnosed in accordance with the clinical guidelines of the Japan Society of Obstetrics and Gynecology [33, 34]. In this cross-sectional study, gestational hypertension and diabetes mellitus were treated as binary variables, recorded as either absent or present. To assess the additive effects of gestational hypertension and diabetes alongside maternal BMI on the risk of childbirth with CL/P, participants were further classified into four groups based on pre-pregnancy overweight status and the presence or absence of gestational hypertension or diabetes.

### Prevalence of CL/P (outcome measure)

Data on CL/P and other congenital anomalies were obtained from medical record transcriptions at delivery and at 1 month of age, as recorded on JECS transcription forms using a list of 61 congenital anomalies with corresponding ICD-10 codes as previously described [5, 30, 35]. The transcription forms included checkboxes for each CL/P type (CL, CLP, and CP), which medical professionals marked upon identifying any orofacial cleft defect, adhering to the common CL/P classification system used worldwide [1, 36]. To avoid potential statistical biases such as overfitting due to small sample size, CL/P, encompassing all phenotypes, was treated as a binary variable in the main analyses. To illustrate the underlying mechanism of an increased risk of CL/P prevalence associated with maternal pre-pregnancy BMI, we also examined differences among CL/P phenotypes (CL, CLP, and CP) and between syndromic and non-syndromic CL/P (presence or absence of other congenital disease[s]), with these results provided in the supplementary material. Mothers with childbirth involving CL/P were categorized into the following groups: CL, CLP, and CP; syndromic and non-syndromic CL/P. These categories were used in logistic regression analyses for multinomial outcomes.

### Covariates

The questionnaire design has been detailed in previous reports [5, 31]. Briefly, sociodemographic, lifestyle, and health status variables of mother–infant dyads, recognized as confounders for maternal nutritional condition, gestational comorbidities, and CL/P prevalence in offspring [28, 29, 35], were included as covariates in the logistic regression models. Data on maternal age at delivery, parity status, infant sex, and prevalence of other congenital diseases (excluding CL/P) were retrieved from medical records. Other variables, including annual household income, maternal educational attainment, smoking, and drinking habits, were assessed via self-administered questionnaires completed by the participating mothers during pregnancy.

Based on the collected data, participants were categorized as follows: infant sex (male or female); parity status (primipara or multipara); annual household income in Japanese yen (<2 million, 2–4 million, 4–6 million, or ≥6 million); maternal educational attainment (junior or high school, junior college/technical college, or university/graduate school); maternal smoking history (never, stopped before or during pregnancy, or currently smoking); maternal alcohol intake (never, stopped before or during pregnancy, or current drinker); and presence or absence of other congenital disease in infants.

### Statistical analysis

Maternal age at delivery is presented as the median with the interquartile range, whereas categorical variables are presented as frequencies and percentages (Table 1). The assumption of “missing at random” was applied to the missing data (Table S1), and multiple imputations by chained equations for continuous and categorical variables were performed using the multivariate normal imputation method with linear and logistic regression models, respectively [37]. An imputation model including all variables used in the main analysis (Tables 1 and S1) was independently applied to 10 copies of the datasets, each with appropriately imputed missing values, in which convergence was estimated by the variance of means and adjusted standard errors among the imputed datasets. According to Rubin’s rules, the imputed values were estimated using means and adjusted standard errors derived from the observed data [38].

**Table 1:** Baseline characteristics on 98373 mother-infant pairs in the JECS

	***CL/P phenotypes, n (%)***				
	**Healthy**	**CL**	**CLP**	**CP**	***p*-value^a^**
	**98118 (99.74)**	**74 (0.08)**	**112 (0.11)**	**69 (0.07)**	
***Age at delivery, median (IQR)***	31 (28, 35)	30 (26, 34)	30 (26, 33)	30 (26, 33)	0.638
***Maternal BMI***					0.098
Normal	71892 (99.76)	52 (0.07)	73 (0.1)	47 (0.07)	
Underweight	16028 (99.74)	12 (0.07)	18 (0.11)	12 (0.07)	
Overweight	10198 (99.60)	10 (0.10)	21 (0.21)	10 (0.10)	
***Gestational hypertension***					<0.001
Absence	95042 (99.75)	72 (0.08)	101 (0.11)	66 (0.07)	
Presence	3077 (99.48)	2 (0.06)	11 (0.36)	3 (0.10)	
***Gestational diabetes mellitus***					0.256
Absence	95108 (99.75)	71 (0.07)	105 (0.11)	67 (0.07)	
Presence	3010 (99.60)	3 (0.10)	7 (0.23)	2 (0.07)	
***Child’s sex***					0.207
Male	50291 (99.72)	40 (0.08)	68 (0.13)	33 (0.07)	
Female	47827 (99.76)	34 (0.07)	44 (0.09)	36 (0.08)	
***Parity status***					0.060
Primipara	40327 (99.74)	21 (0.05)	54 (0.13)	31 (0.08)	
Multipara	57791 (99.74)	53 (0.09)	59 (0.10)	38 (0.07)	
***Household income (million yen/year)***					0.095
<2	5706 (99.68)	6 (0.10)	7 (0.12)	6 (0.10)	
2 to <4	34284 (99.77)	31 (0.09)	35 (0.10)	11 (0.03)	
4 to <6	32292 (99.75)	19 (0.06)	37 (0.11)	26 (0.08)	
≥6	25836 (99.70)	19 (0.07)	33 (0.13)	26 (0.10)	
***Educational attainment***					0.218
High school or lower	35635 (99.72)	24 (0.07)	51 (0.14)	25 (0.07)	
Junior college	41184 (99.75)	36 (0.09)	34 (0.08)	32 (0.08)	
University or higher	21299 (99.75)	14 (0.07)	27 (0.13)	12 (0.06)	
***Smoking habit***					0.470
Never	57033 (99.74)	41 (0.07)	59 (0.10)	46 (0.08)	
Stopped	36352 (99.73)	29 (0.08)	50 (0.14)	21 (0.06)	
Smoking	4733 (99.81)	4 (0.08)	3 (0.07)	2 (0.04)	
***Alcohol intake***					0.595
Never	33873 (99.73)	29 (0.09)	40 (0.12)	24 (0.07)	
Stopped	54562 (99.74)	42 (0.08)	65 (0.12)	38 (0.07)	
Drinking	9683 (99.82)	3 (0.03)	7 (0.07)	7 (0.07)	
***Other congenital diseases***					<0.001
Absence	89802 (99.79)	67 (0.07)	81 (0.09)	43 (0.05)	
Presence	8316 (99.24)	7 (0.08)	31 (0.37)	26 (0.31)	

In some cases, the number of imputed variables does not match the total because of rounding to 0 or 1 (based on a cutoff of 0.5)

^a^Analysis of variance for continuous variables or Pearson’s chi-square test for categorical variables by CL/P phenotype, with *p*-values indicating statistical significance across groups.

Abbreviations: BMI: body mass index; CL: cleft lip; CL/P: cleft lip and/or palate; CLP: cleft lip and palate; CP: isolated cleft palate; IQR: interquartile range; JECS: Japan Environment and Children’s Study.

Using the control group as a reference, crude and multivariate logistic regression analyses were conducted to estimate the odds ratios (ORs) for the association between maternal BMI, gestational hypertension, diabetes, and their comorbid conditions with childbirth involving CL/P. For crude or adjusted analyses using the covariates, Model 1 was analyzed after adjusting for maternal age at delivery, parity status, and the infant’s sex. Model 2 included maternal factors (educational attainment, smoking and drinking habits), household income, and prevalence of other congenital diseases, in addition to the variables included in Model 1. Model 3 additionally included the prevalence of gestational comorbidities. Complete-case analyses, using the dataset comprising only cases without missing values (Table S2), were also conducted as sensitivity analyses. Additionally, to avoid statistical bias because of the small sample size in subgroup analyses, the propensity scores of each individual were calculated using a logistic regression model for CL/P prevalence and applied as a single covariate for a simplified adjustment. All statistical analyses were performed using SPSS software (version 24.0; IBM Corp., Armonk, NY, USA). Statistical significance was set at *p* < 0.05.

## Results

Participant characteristics are presented in Table 1. Of the 255 infants with CL/P (overall prevalence: 0.26%), 112 (43.9%) had CLP, 74 (29.0%) had CL, and 69 (27.1%) had CP. Among them, 64 infants (25.1%) were diagnosed with syndromic CL/P. Of these, CP (40.6%) and CL (10.9%) phenotypes had a higher and lower prevalence, respectively, than the rates in the entire study CL/P population. The mean (standard deviation) pre-pregnancy BMI in the control and CL/P groups was 21.2 (3.3) kg/m^2^ and 21.6 (3.8) kg/m^2^, respectively. Furthermore, 10,239 maternal participants (10.4%) were classified as overweight (BMI ≥ 25.0 kg/m^2^), including 2,339 (2.4%) who were obese (BMI > 30.0 kg/m^2^). Owing to the small sample size and the general BMI distribution among Japanese women [26], obese participants were included in the overweight group.

The crude and adjusted ORs with 95% confidence intervals (CIs) for the association between pre-pregnancy BMI and childbirth with CL/P are presented in Table 2. In Model 3, which included all covariates, the adjusted ORs (95% CIs, *p*-values) for childbirth with CL/P among underweight and overweight participants were 1.08 (0.81–1.43, *p* = 0.670) and 1.58 (1.11–2.24, *p* = 0.011), respectively, compared with normal BMI participants. Participant characteristics and the results of the complete-case analysis are shown in Tables S2 and S3, respectively. Similarly, the adjusted ORs for childbirth with CL/P was significantly increased in participating mothers with overweight, but not in those with underweight (Table S3). In addition, we examined the crude and adjusted ORs (95% CIs) of pre-pregnancy maternal BMI for childbirth with each CL/P phenotype (Table S4: CL, CLP, and CP) and for cases with other congenital comorbidities (Table S5: syndromic or non-syndromic CL/P and other congenital diseases without CL/P). After adjusting for all covariates, the adjusted ORs (95% CIs, *p*-values) for childbirth with CL, CLP, and CP among participants with pre-pregnancy overweight were 1.30 (0.65–2.59, *p* = 0.463), 1.79 (1.08–2.97, *p* = 0.025), and 1.55 (0.77–3.10, *p* = 0.222), respectively. No significant associations were observed between pre-pregnancy underweight and individual CL/P phenotypes. For non-syndromic and syndromic CL/P, the ORs associated with pre-pregnancy overweight were 1.69 (1.13–2.52, *p* = 0.010) and 1.23 (0.58–2.61, *p* = 0.586), respectively. When participants were stratified by gestational hypertension and diabetes mellitus as exposure measures, the adjusted ORs (95% CIs, *p*-values) for childbirth with CL/P was significantly increased in participating mothers with gestational hypertension (1.80, 95% CI: 1.07–3.02, *p* = 0.027), but not in those with gestational diabetes (1.36, 95% CI: 0.75–2.46, *p* = 0.312) (Table S6).

**Table 2:** Association between pre-pregnancy BMI and risk of orofacial clefts in offspring

	**Normal**	**Underweight**	***p*-value**	**Overweight**	***p*-value**
**CL/P prevalence, n (%)**	**172 (0.24)**	**42 (0.26)**		**41 (0.40)**	
Crude	Ref	1.10 (0.78–1.54)	0.597	1.68 (1.20–2.36)	0.003
Model 1^a^		1.08 (0.91–1.29)	0.642	1.69 (1.23–2.32)	0.003
Model 2^b^		1.07 (0.83–1.39)	0.697	1.68 (1.19–2.37)	0.003
Model 3^c^		1.08 (0.81–1.43)	0.670	1.58 (1.11–2.24)	0.011

Data are presented as odds ratios (95% confidence intervals).

^a^Adjusted for maternal age, parity status, and child’s sex.

^b^Additionally adjusted for maternal educational attainment, smoking and drinking habits, household income, and prevalence of other congenital diseases (based on Model 1).

^c^Additionally adjusted for gestational hypertension and gestational diabetes (based on Model 2).

Abbreviations: BMI: body mass index; CL/P: cleft lip and/or palate; Ref: reference category.

Furthermore, we analyzed the interaction between pre-pregnancy overweight status and the prevalence of gestational comorbidities in relation to childbirth with CL/P. Compared with the reference group (healthy participants with a normal pre-pregnancy BMI [<25.0]), the OR (95% CI, *p*-value) for CL/P in offspring of overweight mothers with gestational hypertension was 2.91 (1.28–6.61, *p* = 0.011), whereas the OR for those with gestational diabetes was 2.12 (0.87–5.19, *p* = 0.100) (Table 3). The ORs in the analyses using the complete-case dataset and the propensity scores of individuals are shown in Tables S7 and S8, respectively. Similarly, the adjusted ORs for childbirth with CL/P was significantly increased in overweight mothers, particularly with gestational hypertension (Tables S7 and S8).

**Table 3:** Association between maternal pre-pregnancy BMI, gestational comorbidities, and orofacial cleft defects in infants

***Overweight***	**−**	**+**		**−**		**+**	

***Hypertension***	**−**	**−**		**+**		**+**	
**CL/P prevalence, n (%)**	204 (0.24)	35 (0.37)	*p*-value	10 (0.45)	*p*-value	6 (0.70)	*p*-value
Crude	Ref	1.57 (1.31–1.89)	0.013	1.89 (1.36–2.61)	0.051	2.97 (1.96–4.50)	0.009
Model 1^a^		1.58 (1.10–2.26)	0.013	1.92 (1.01–3.64)	0.046	3.03 (1.34–6.85)	0.008
Model 2^b^		1.58 (1.10–2.27)	0.013	1.77 (0.93–3.36)	0.081	2.91 (1.28–6.61)	0.011

***Diabetes mellitus***	**−**	**−**		**+**		**+**	

**CL/P prevalence, n (%)**	207 (0.24)	36 (0.39)	*p*-value	7 (0.34)	*p*-value	5 (0.53)	*p*-value
Crude	Ref	1.61 (1.34–1.93)	0.009	1.40 (0.95–2.06)	0.384	2.22 (1.41–3.50)	0.079
Model 1^a^		1.62 (1.14–2.30)	0.008	1.43 (0.82–2.49)	0.352	2.27 (1.44–3.58)	0.071
Model 2^b^		1.63 (1.14–2.33)	0.007	1.39 (0.66–2.94)	0.391	2.12 (0.87–5.19)	0.100

Data are presented as odds ratios (95% confidence intervals).

^a^Adjusted for maternal age, parity status, and child’s sex.

^b^Additionally adjusted for maternal educational attainment, smoking and drinking habits, household income, and prevalence of other congenital diseases (based on Model 1).

Abbreviations: BMI: body mass index; CL/P: cleft lip and/or palate; Ref: reference category.

## Discussion

The key finding of this study, which utilized a nationwide dataset from a prospective birth cohort in Japan, was that maternal pre-pregnancy overweight, but not underweight, was associated with increased CL/P prevalence in offspring. Gestational disorders, particularly hypertension, combined with maternal overweight further increased the CL/P risk. Our results suggest that a better understanding of CL/P pathogenesis can provide valuable insights for promoting pre- and inter-conceptional care.

In our results from a large-scale Japanese birth cohort study, pre-pregnancy overweight (BMI ≥ 25.0 kg/m^2^) was significantly associated with a higher prevalence of CL/P in offspring, whereas underweight (BMI < 18.5 kg/m^2^) was not. The impact of overweight and obesity on CL/P risk is well documented [13–17], whereas the association between underweight and CL/P prevalence has been debated [13–16]. Given that CL/P prevalence varies across geographic regions and racial backgrounds in Japan [3, 4], the approximately 1.5-fold increased risk observed among overweight mothers in our study (Table 2) is slightly higher than the risks reported in previous meta-analyses [12, 16, 17]. Among the different CL/P classifications (CL, CLP, and CP) and genetic categories (syndromic and non-syndromic CL/P), maternal overweight, but not underweight, was predominantly associated with CLP (Table S4) and non-syndromic CL/P (Table S5). Regarding maternal underweight and offspring CL/P, maternal micronutrient intake during the first trimester did not differ between mothers with and without offspring affected by CL/P, as reported by Yoshida et al. [39] using the JECS dataset. Thus, underweight participants in this study likely did not experience malnutrition, including folate or vitamin deficiencies, which increase CL/P risk [40, 41]. However, underweight in pregnant women is associated with an increased risk of congenital anomalies in their offspring [14, 16]; thus, further research is needed to clarify the relationship between maternal underweight and congenital anomalies to better understand the underlying mechanisms.

Both gestational hypertension and diabetes mellitus, two of the most common comorbidities encountered in pregnant women, especially those who are overweight or obese, have been implicated as possible causal factors for CL/P and other congenital anomalies [14, 17, 42]. In this study (Tables 3 and S6), gestational hypertension was independently associated with a higher prevalence of CL/P in offspring, whereas diabetes mellitus appeared to contribute synergistically to the risk when combined with pre-pregnancy overweight. Syndromic CL/P is typically associated with defined genetic syndromes or chromosomal anomalies, whereas non-syndromic CL/P results from multifactorial influences, combining genetic predisposition with environmental exposures [43]. The underlying pathophysiological mechanisms linking increased CL/P prevalence in obese women with gestational comorbidities are likely multifactorial, involving metabolic abnormalities, hormonal imbalance, systemic inflammation, oxidative stress, and genomic damage [16, 17, 42]. In mouse models, experimental hypoxia during pregnancy increases the CL/P incidence in offspring [44, 45]. Supporting this, Åmark et al. [19] reported an increased risk of fetal hypoxia–ischemia in infants born to mothers who had gestational complications, such as obesity, hypertension, and diabetes, which are associated with endothelial dysfunction. In conclusion, the complicated causal factors underlying CL/P pathogenesis require systematic investigation by breaking them down into smaller components. Maintaining an appropriate pre-pregnancy BMI is essential for a healthy pregnancy onset and, based on our results, may be particularly relevant to a decreased risk of CL/P occurrence. Pre-conception counseling and regular prenatal checkups focused on body weight control and the adequate management of nutritional balance and gestational comorbidities before and during pregnancy may be essential in improving maternal and neonatal outcomes.

This study has several advantages as well as some limitations worth noting. First, the JECS dataset did not include information on prenatal management of maternal weight, gestational hypertension, or diabetes mellitus, leaving clinical histories of gestational disorders during pregnancy unknown. However, the JECS dataset, covering approximately 45% of all live births within the study area, provided a large control population, enhancing the generalizability with statistical power [46]. Despite the large sample size of nearly 100,000 mother-infant pairs, approximately 250 children with CL/P were included in the analysis. Although it is an inherent constraint of rare disease studies, the small sample sizes in some subgroups (Table 3) may cause statistical concerns such as overfitting. Consequently, the relatively small number of CL/P cases limits the reproducibility and generalizability of our findings. Future studies with larger numbers of affected participants are warranted.

## Conclusion

High maternal BMI before pregnancy was associated with an increased risk of CL/P in offspring. In particular, comorbid gestational hypertension tended to further elevate this risk. Focused on the key findings, nutritional management can empower pregnant women to achieve healthier outcomes for both mother and child.

## Abbreviations

BMI: body mass index
CI: confidence interval
CL: cleft lip
CL/P: cleft lip and/or cleft palate
CLP: cleft lip with palate
CP: isolated cleft lip
JECS: Japan Environment and Children’s Study
OR: odds ratio

## Supplementary information

**Additional file 1:** **Table S1** Baseline characteristics of missing data among 98373 mother–infant pairs participating in the JECS. **Table S2** Baseline characteristics of 87182 mother–infant pairs in the complete dataset. **Table S3** Association between pre-pregnancy BMI and risk of orofacial clefts in offspring according to the complete-case analysis. **Table S4** Association between maternal BMI and childbirth outcomes by individual phenotypes of orofacial cleft defects. **Table S5** Association between maternal BMI and childbirth with syndromic or non-syndromic orofacial cleft defects. **Table S6** Association of gestational comorbidities with orofacial cleft defects. **Table S7** Association between maternal pre-pregnancy BMI, gestational comorbidities, and orofacial cleft defects in infants according to the complete-case analysis. **Table S8** Association between maternal pre-pregnancy BMI, gestational comorbidities, and orofacial cleft defects in infants using the propensity score of each individual.

## Declarations

### Availability of data and material

Data are unsuitable for public deposition, owing to ethical restrictions and the legal framework of Japan. It is prohibited by the Act on the Protection of Personal Information (Act No. 57 of May 30, 2003, amended on September 9, 2015) to publicly deposit data containing personal information. Ethical Guidelines for Medical and Health Research Involving Human Subjects enforced by the Japan Ministry of Education, Culture, Sports, Science and Technology and the Ministry of Health, Labour and Welfare also restrict the open sharing of epidemiological data. All inquiries about access to data were sent to jecs-en@nies.go.jp. The person responsible for handling inquiries sent to this e-mail address is Dr. Shoji F. Nakayama, JECS Program Office, National Institute for Environmental Studies.
