# Supplementary material for: Impact of maternal body mass index and gestational comorbidities on the birth prevalence of orofacial clefts in the Japan Environment and Children’s Study
Source: Environ Health Prev Med. 2025 Nov 1;30:86. doi: 10.1265/ehpm.25-00205 (PMC12611479; doi:10.1265/ehpm.25-00205)
Supplement: Supplementary file 1 — Additional file 1: Table S1 Baseline characteristics of missing data among 98373 mother–infant pairs participating in the JECS. Table S2 Baseline characteristics of 87182 mother–infant pairs in the complete dataset. Table S3 Association between pre-pregnancy BMI and risk of orofacial clefts in offspring according to the complete-case analysis. Table S4 Association between maternal BMI and childbirth outcomes by individual phenotypes of orofacial cleft defects. Table S5 Association between maternal BMI and childbirth with syndromic or non-syndromic orofacial cleft defects. Table S6 Association of gestational comorbidities with orofacial cleft defects. Table S7 Association between maternal pre-pregnancy BMI, gestational comorbidities, and orofacial cleft defects in infants according to the complete-case analysis. Table S8 Association between maternal pre-pregnancy BMI, gestational comorbidities, and orofacial cleft defects in infants using the propensity score of each individual. [file ehpm-30-086-s001.docx]

| **Table S1** Baseline characteristics of missing data among 98373 mother–infant pairs participating in the JECS | | |
| --- | --- | --- |
| **Missing, n (%)** | **Control** | **CL/P** |
| **Congenital disease** | 0 (0.00) | 0 (0.00) |
| **Maternal age at delivery** | 10 (0.01) | 0 (0.00) |
| **Pre-pregnancy BMI** | 0 (0.00) | 0 (0.00) |
| **Gestational hypertension** | 285 (0.29) | 0 (0.00) |
| **Gestational diabetes** | 285 (0.29) | 0 (0.00) |
| **Child’s sex** | 18 (0.02) | 0 (0.00) |
| **Parity status** | 2332 (2.39) | 10 (3.91) |
| **Household income (million yen/year)** | 8565 (8.73) | 25 (9.80) |
| **Educational attainment** | 2213 (2.26) | 7 (2.75) |
| **Smoking habit** | 915 (0.93) | 1 (0.39) |
| **Alcohol intake** | 786 (0.80) | 1 (0.39) |
| Abbreviations: BMI: body mass index; CL/P: cleft lip and/or palate; JECS: Japan Environment and Children’s Study. | | |

| **Table S2** Baseline characteristics of 87182 mother–infant pairs in the complete dataset | | | | | | | | | | | | | | | | | | | | | |
| --- | --- | --- | --- | --- | --- | --- | --- | --- | --- | --- | --- | --- | --- | --- | --- | --- | --- | --- | --- | --- | --- |
|  | | ***CL/P phenotypes, n (%)*** | | | | | | | | | | | | | | | | | | | |
|  | | **Healthy** | | | | **CL** | | | | **CLP** | | | | **CP** | | | | |  | | |
|  | | **86963 (99.75)** | | | | **62 (0.07)** | | | | **96 (0.11)** | | | | **61 (0.07)** | | | | | ***p-*value^a^** | | |
| ***Age at delivery, median (IQR)*** | | 31 (28, 35) | | | 33 (28, 35) | | | | 31 (27, 34) | | | | 32 (28, 35) | | | | | 0.889 | | |  |
| ***Maternal BMI*** | |  | | |  | | | |  | | | |  | | | | | 0.109 | | |  |
| Normal | | 63999 (99.77) | | | 43 (0.07) | | | | 61 (0.10) | | | | 43 (0.07) | | | | |  | | |  |
| Underweight | | 13947 (99.74) | | | 10 (0.07) | | | | 17 (0.12) | | | | 9 (0.06) | | | | |  | | |  |
| Overweight | | 9017 (99.6) | | | 9 (0.10) | | | | 18 (0.20) | | | | 9 (0.10) | | | | |  | | |  |
| ***Gestational hypertension*** | | | | | | | | | | | | | | | | 0.001 | | | |  |  |
| Absence | 84274 (99.76) | | 60 (0.07) | | | | 86 (0.10) | | | | 58 (0.07) | | | | |  | | | |  |  |
| Presence | 2689 (99.45) | | 2 (0.07) | | | | 10 (0.37) | | | | 3 (0.11) | | | | |  | | | |  |  |
| ***Gestational diabetes mellitus*** | | | | | | | | | | | | | | | | 0.134 | | | |  |  |
| Absence | 84260 (99.75) | | 60 (0.07) | | | | 89 (0.11) | | | | 59 (0.07) | | | | |  | | | |  |  |
| Presence | 2703 (99.59) | | 2 (0.07) | | | | 7 (0.26) | | | | 2 (0.07) | | | | |  | | | |  |  |
| ***Child's sex*** |  | |  | | | |  | | | |  | | | | | 0.550 | | | |  |  |
| Male | 44456 (99.73) | | 33 (0.07) | | | | 56 (0.13) | | | | 31 (0.07) | | | | |  | | | |  |  |
| Female | 42507 (99.77) | | 29 (0.07) | | | | 40 (0.09) | | | | 30 (0.07) | | | | |  | | | |  |  |
| ***Parity status*** |  | |  | | | |  | | | |  | | | | | 0.183 | | | |  |  |
| Primipara | 35376 (99.75) | | 18 (0.05) | | | | 43 (0.12) | | | | 28 (0.08) | | | | |  | | | |  |  |
| Multipara | 51587 (99.75) | | 44 (0.09) | | | | 53 (0.10) | | | | 33 (0.06) | | | | |  | | | |  |  |
| ***Household income (million yen/year)*** | | | | | | | | | | | | | | | | 0.167 | | | | |  |
| < 2 | 4883 (99.71) | | 4 (0.08) | | | | 6 (0.12) | | | | 4 (0.08) | | | | |  | | | |  |  |
| 2 to < 4 | 29965 (99.79) | | 25 (0.08) | | | | 27 (0.09) | | | | 10 (0.03) | | | | |  | | | |  |  |
| 4 to < 6 | 28788 (99.75) | | 16 (0.06) | | | | 33 (0.11) | | | | 24 (0.08) | | | | |  | | | |  |  |
| ≥ 6 | 23327 (99.70) | | 17 (0.07) | | | | 30 (0.13) | | | | 23 (0.10) | | | | |  | | | |  |  |
| ***Educational attainment*** | | | | | | | | | | | | | | | | | 0.191 | | | |  |
| High school or lower | 30666 (99.73) | | 20 (0.07) | | | | 42 (0.14) | | | | 21 (0.07) | | | | |  | | | |  |  |
| Junior college | 36819 (99.76) | | 30 (0.08) | | | | 28 (0.08) | | | | 29 (0.08) | | | | |  | | | |  |  |
| University or higher | 19478 (99.75) | | 12 (0.06) | | | | 26 (0.13) | | | | 11 (0.06) | | | | |  | | | |  |  |
| ***Smoking habit*** | |  | |  | | | |  | | | |  | | | 0.809 | | | | |  |  |
| Never | | 50862 (99.75) | | 36 (0.07) | | | | 54 (0.11) | | | | 39 (0.08) | | |  | | | | |  |  |
| Stopped | | 32052 (99.74) | | 22 (0.07) | | | | 39 (0.12) | | | | 21 (0.07) | | |  | | | | |  |  |
| Smoking | | 4049 (99.8) | | 4 (0.10) | | | | 3 (0.07) | | | | 1 (0.02) | | |  | | | | |  |  |
| ***Alcohol intake*** | |  | |  | | | |  | | | |  | | | 0.649 | | | | |  |  |
| Never | | 29880 (99.74) | | 23 (0.08) | | | | 34 (0.11) | | | | 21 (0.07) | | |  | | | | |  |  |
| Stopped | | 48289 (99.74) | | 37 (0.08) | | | | 55 (0.11) | | | | 33 (0.07) | | |  | | | | |  |  |
| Drinking | | 8794 (99.82) | | 2 (0.02) | | | | 7 (0.08) | | | | 7 (0.08) | | |  | | | | |  |  |
| ***Other congenital diseases*** | |  | |  | | | |  | | | |  | | | < 0.001 | | | | |  |  |
| Absence | | 79523 (99.79) | | 57 (0.07) | | | | 70 (0.09) | | | | 39 (0.05) | | |  | | | | |  |  |
| Presence | | 7440 (99.29) | | 5 (0.07) | | | | 26 (0.35) | | | | 22 (0.29) | | |  | | | | |  |  |
| ^a^Analysis of variance for continuous variables or Pearson’s chi-square test for categorical variables by CL/P phenotypes, with *p*-values indicating statistical significance across groups.  Abbreviations: BMI: body mass index; CL: cleft lip; CL/P: cleft lip and/or palate; CLP: cleft lip and palate; CP: isolated cleft palate; IQR: interquartile range; JECS: Japan Environment and Children’s Study. | | | | | | | | | | | | | | | | | | | | | |

| **Table S3** Association between pre-pregnancy BMI and risk of orofacial clefts in offspring according to the complete-case analysis | | | | | |
| --- | --- | --- | --- | --- | --- |
|  | **Normal** | **Underweight** | ***p-*value** | **Overweight** | ***p-*value** |
| **CL/P prevalence, n (%)** | 147 (0.23) | 36 (0.26) |  | 36 (0.40) |  |
| Crude | Ref | 1.12 (0.78–1.62) | 0.531 | 1.74 (1.21–2.50) | 0.003 |
| Model 1^a^ |  | 1.12 (0.78–1.62) | 0.543 | 1.74 (1.21–2.51) | 0.003 |
| Model 2^b^ |  | 1.11 (0.77–1.60) | 0.588 | 1.74 (1.21–2.52) | 0.003 |
| Model 3^c^ |  | 1.12 (0.77–1.61) | 0.557 | 1.61 (1.11–2.35) | 0.013 |
| Data are presented as odds ratios (95% confidence intervals). | | | | | |
| ^a^Adjusted for maternal age, parity status, and child’s sex. | | | | | |
| ^b^Additionally adjusted for maternal educational attainment, smoking and drinking habits, household income, and prevalence of other congenital diseases (based on Model 1). | | | | | |
| ^c^Additionally adjusted for gestational hypertension and gestational diabetes (based on Model 2). | | | | | |
| Abbreviations: BMI: body mass index; CL/P: cleft lip and/or palate; Ref: reference category. | | | | | |

| **Table S4** Association between maternal BMI and childbirth outcomes by individual phenotypes of orofacial cleft defects | | | | | | |
| --- | --- | --- | --- | --- | --- | --- |
|  | | | | | |  |
| **CL** | **Normal** | **Underweight** | ***p-*value** | **Overweight** | ***p-*value** | |
| **Prevalence, n (%)** | 52 (0.07) | 12 (0.07) |  | 10 (0.10) |  | |
| Crude | Ref | 1.04 (0.55–1.94) | 0.914 | 1.36 (0.69–2.67) | 0.378 | |
| Model 1^a^ |  | 1.04 (0.76–1.44) | 0.898 | 1.32 (0.93–1.86) | 0.423 | |
| Model 2^b^ |  | 1.03 (0.75–1.42) | 0.928 | 1.31 (0.66–2.59) | 0.439 | |
| Model 3^c^ |  | 1.03 (0.75–1.42) | 0.927 | 1.30 (0.65–2.59) | 0.463 | |
| **CLP** | **Normal** | **Underweight** | ***p-*value** | **Overweight** | ***p-*value** | |
| **Prevalence, n (%)** | 73 (0.10) | 18 (0.11) |  | 21 (0.21) |  | |
| Crude | Ref | 1.11 (0.66–1.85) | 0.702 | 2.03 (1.25–3.30) | 0.004 | |
| Model 1^a^ |  | 1.08 (0.83–1.40) | 0.786 | 2.07 (1.28–3.36) | 0.003 | |
| Model 2^b^ |  | 1.06 (0.62–1.80) | 0.826 | 2.05 (1.25–3.34) | 0.004 | |
| Model 3^c^ |  | 1.07 (0.66–1.74) | 0.790 | 1.79 (1.08–2.97) | 0.025 | |
| **CP** | **Normal** | **Underweight** | ***p-*value** | **Overweight** | ***p-*value** | |
| **Prevalence, n (%)** | 47 (0.07) | 12 (0.07) |  | 10 (0.10) |  | |
| Crude | Ref | 1.15 (0.61–2.16) | 0.675 | 1.50 (0.76–2.97) | 0.245 | |
| Model 1^a^ |  | 1.15 (0.83–1.59) | 0.673 | 1.51 (0.76–3.00) | 0.236 | |
| Model 2^b^ |  | 1.13 (0.57–2.26) | 0.705 | 1.54 (0.77–3.05) | 0.223 | |
| Model 3^c^ |  | 1.13 (0.69–1.86) | 0.705 | 1.55 (0.77–3.10) | 0.222 | |
| Data are presented as odds ratios (95% confidence intervals). | | | | | | |
| ^a^Adjusted for maternal age, parity status, and child’s sex. | | | | | | |
| ^b^Additionally adjusted for maternal educational attainment, smoking and drinking habits, household income, and prevalence of other congenital diseases (based on Model 1). | | | | | | |
| ^c^Additionally adjusted for gestational hypertension and gestational diabetes (based on Model 2). | | | | | | |
| Abbreviations: BMI: body mass index; CL: cleft lip only; CLP: cleft lip with palate; CP: cleft palate only; Ref: reference category. | | | | | | |

| **Table S5** Association between maternal BMI and childbirth with syndromic or non-syndromic orofacial cleft defects  **Table S3: Association between maternal BMI and childbirth with syndromic and non-syndromic orofacial cleft defects** | | | | | |
| --- | --- | --- | --- | --- | --- |
|  | | | | | |
| **Non-syndromic CL/P** | **Normal** | **Underweight** |  | **Overweight** |  |
| **Prevalence, n (%)** | 130 (0.18) | 29 (0.18) | *p-*value | 32 (0.31) | *p-*value |
| Crude | Ref | 1.00 (0.67–1.50) | 0.985 | 1.74 (1.18–2.57) | 0.005 |
| Model 1^a^ |  | 0.99 (0.81–1.22) | 0.958 | 1.75 (1.44–2.14) | 0.005 |
| Model 2^b^ |  | 0.98 (0.80–1.21) | 0.927 | 1.75 (1.18–2.59) | 0.005 |
| Model 3^c^ |  | 0.99 (0.80–1.21) | 0.944 | 1.69 (1.13–2.52) | 0.010 |
| **Syndromic CL/P** | **Normal** | **Underweight** |  | **Overweight** |  |
| **Prevalence, n (%)** | 42 (0.06) | 13 (0.08) | *p-*value | 9 (0.09) | *p-*value |
| Crude | Ref | 1.39 (0.75–2.60) | 0.296 | 1.52 (0.74–3.12) | 0.256 |
| Model 1^a^ |  | 1.41 (1.02–1.94) | 0.283 | 1.51 (0.74–3.10) | 0.263 |
| Model 2^b^ |  | 1.32 (0.71–2.47) | 0.386 | 1.45 (0.70–3.00) | 0.318 |
| Model 3^c^ |  | 1.33 (0.71–2.49) | 0.375 | 1.23 (0.58–2.61) | 0.586 |
| Data are presented as odds ratios (95% confidence intervals). | | | | | |
| ^a^Adjusted for maternal age, parity status, and child’s sex. | | | | | |
| ^b^Additionally adjusted for maternal educational attainment, smoking and drinking habits, household income, and prevalence of other congenital diseases (based on Model 1). | | | | | |
| ^c^Additionally adjusted for gestational hypertension and gestational diabetes mellitus (based on Model 2). | | | | | |
| Abbreviations: BMI: body mass index; CL/P: cleft lip and/or palate; Ref: reference category. | | | | | |

| **Table S6** Association of gestational comorbidities with orofacial cleft defects | | | |  |
| --- | --- | --- | --- | --- |
|  | **Control** | **CL/P** |  | |
| **Hypertension prevalence, n (%)** | **239 (0.25)** | **16 (0.52)** | ***p-*value** | |
| Crude | Ref | 2.07 (1.60–2.68) | 0.005 | |
| Model 1^a^ |  | 2.11 (1.27–3.52) | 0.004 | |
| Model 2^b^ |  | 1.96 (1.17–3.27) | 0.010 | |
| Model 3^c^ |  | 1.80 (1.07–3.02) | 0.027 | |
|  | **Control** | **CL/P** |  | |
| **Diabetes mellitus prevalence, n (%)** | 243 (0.25) | 12 (0.40) | *p-*value | |
| Crude | Ref | 1.56 (1.16–2.10) | 0.133 | |
| Model 1^a^ |  | 1.60 (0.93–2.73) | 0.117 | |
| Model 2^b^ |  | 1.53 (0.85–2.74) | 0.157 | |
| Model 3^c^ |  | 1.36 (0.75–2.46) | 0.312 | |
| Data are presented as odds ratios (95% confidence intervals). | | | | |
| ^a^Adjusted for maternal age, parity status, and child's sex. | | | | |
| ^b^Additionally adjusted for maternal educational attainment, smoking and drinking habits, household income, and prevalence of other congenital diseases (based on Model 1). | | | | |
| ^c^Additionally adjusted for maternal BMI before pregnancy (based on Model 2). | | | | |
| Abbreviations: BMI: body mass index; CL/P: cleft lip and/or palate; Ref: reference category. | | | | |

| **Table S7** Association between maternal pre-pregnancy BMI, gestational comorbidities, and orofacial cleft defects in infants according to the complete-case analysis | | | | | | | |
| --- | --- | --- | --- | --- | --- | --- | --- |
|  | | | | | | | |
| ***Overweight*** | **-** | **+** |  | **-** |  | **+** |  |
| ***Hypertension*** | **-** | **-** |  | **+** |  | **+** |  |
| **CL/P prevalence, n (%)** | 174 (0.23) | 30 (0.36) | *p-*value | 9 (0.46) | *p-*value | 6 (0.81) | *p-*value |
| Crude | Ref | 1.58 (1.07–2.33) | 0.021 | 2.01 (1.03–3.94) | 0.041 | 3.56 (1.57–8.06) | 0.002 |
| Model 1^a^ |  | 1.58 (1.07–2.33) | 0.021 | 2.04 (1.04–4.00) | 0.039 | 3.60 (1.59–8.16) | 0.002 |
| Model 2^b^ |  | 1.59 (1.08–2.36) | 0.020 | 1.90 (0.97–3.75) | 0.062 | 3.53 (1.55–8.04) | 0.003 |
| ***Diabetes mellitus*** | **-** | **-** |  | **+** |  | **+** |  |
| **CL/P prevalence, n (%)** | 177 (0.23) | 31 (0.38) | *p-*value | 6 (0.32) | *p-*value | 5 (0.60) | *p-*value |
| Crude | Ref | 1.63 (1.11–2.38) | 0.013 | 1.38 (0.61–3.11) | 0.444 | 2.60 (1.07–6.34) | 0.036 |
| Model 1^a^ |  | 1.63 (1.11–2.39) | 0.012 | 1.39 (0.61–3.14) | 0.434 | 2.62 (1.07–6.39) | 0.035 |
| Model 2^b^ |  | 1.65 (1.12–2.42) | 0.011 | 1.36 (0.60–3.08) | 0.463 | 2.46 (1.00–6.04) | 0.049 |
| Data are presented as odds ratios (95% confidence intervals). | | | | | | | |
| ^a^Adjusted for maternal age, parity status, and child’s sex. | | | | | | | |
| ^b^Additionally adjusted for maternal educational attainment, smoking and drinking habits, household income, and prevalence of other congenital diseases (based on Model 1). | | | | | | | |
| Abbreviations: BMI: body mass index; CL/P: cleft lip and/or palate. | | | | | | | |

| **Table S8** Association between maternal pre-pregnancy BMI, gestational comorbidities, and orofacial cleft defects in infants using the propensity score of each individual | | | | | | | |
| --- | --- | --- | --- | --- | --- | --- | --- |
|  | | | | | | | |
| ***Overweight*** | **-** | **+** |  | **-** |  | **+** |  |
| ***Hypertension*** | **-** | **-** |  | **+** |  | **+** |  |
| **CL/P prevalence, n (%)** | 204 (0.24) | 35 (0.37) | *p-*value | 10 (0.45) | *p-*value | 6 (0.70) | *p-*value |
| Crude | Ref | 1.57 (1.31–1.89) | 0.013 | 1.89 (1.36–2.61) | 0.051 | 2.97 (1.96–4.50) | 0.009 |
| Model 1^a^ |  | 1.58 (1.10–2.26) | 0.013 | 1.91 (1.01–3.61) | 0.046 | 3.01 (1.34–6.80) | 0.008 |
| Model 2^b^ |  | 1.57 (1.09–2.25) | 0.014 | 1.75 (0.93–3.30) | 0.086 | 2.87 (1.27–6.49) | 0.011 |
| ***Diabetes mellitus*** | **-** | **-** |  | **+** |  | **+** |  |
| **CL/P prevalence, n (%)** | 207 (0.24) | 36 (0.39) | *p-*value | 7 (0.34) | *p-*value | 5 (0.53) | *p-*value |
| Crude | Ref | 1.61 (1.34–1.93) | 0.009 | 1.40 (0.95–2.06) | 0.384 | 2.22 (1.41–3.50) | 0.079 |
| Model 1^a^ |  | 1.62 (1.14–2.30) | 0.008 | 1.43 (0.97–2.10) | 0.356 | 2.26 (1.44–3.56) | 0.072 |
| Model 2^b^ |  | 1.61 (1.13–2.30) | 0.008 | 1.39 (0.65–2.95) | 0.398 | 2.10 (0.86–5.12) | 0.102 |
| Data are presented as odds ratios (95% confidence intervals). | | | | | | | |
| ^a^Adjusted using individuals’ propensity scores from maternal age, parity status, and child’s sex. | | | | | | | |
| ^b^Additionally adjusted using individuals’ propensity scores from maternal educational attainment, smoking and drinking habits, household income, and prevalence of other congenital diseases (based on Model 1). | | | | | | | |
| Abbreviations: BMI: body mass index; CL/P: cleft lip and/or palate. | | | | | | | |
